# Comparisons of photosynthesis‐related traits of 27 abundant or subordinate bryophyte species in a subalpine old‐growth fir forest

**DOI:** 10.1002/ece3.3277

**Published:** 2017-08-11

**Authors:** Zhe Wang, Maaike Y. Bader, Xin Liu, Zhangming Zhu, Weikai Bao

**Affiliations:** ^1^ CAS Key Laboratory of Mountain Ecological Restoration and Bioresource Utilization & Ecological Restoration and Biodiversity Conservation Key Laboratory of Sichuan Province Chengdu Institute of Biology Chinese Academy of Sciences Chengdu China; ^2^ College of Life and Environmental Sciences Shanghai Normal University Shanghai China; ^3^ Ecological Plant Geography Faculty of Geography University of Marburg Marburg Germany; ^4^ School of Ecology and Environmental science Yunnan University Kunming China

**Keywords:** adaptation, competition, dominance, ecophysiology, feather moss, functional traits, gas exchange, growth form

## Abstract

Bryophyte communities can exhibit similar structural and taxonomic diversity as vascular plant communities, just at a smaller scale. Whether the physiological diversity can be similarly diverse, and whether it can explain local abundance patterns is unknown, due to a lack of community‐wide studies of physiological traits. This study re‐analyzed data on photosynthesis‐related traits (including the nitrogen, phosphorus and chlorophyll concentrations, photosynthetic capacities, and photosynthetic nutrient use efficiencies) of 27 bryophyte species in a subalpine old‐growth fir forest on the eastern Tibetan Plateau. We explored differences between taxonomic groups and hypothesized that the most abundant bryophyte species had physiological advantages relative to other subdominant species. Principal component analysis (PCA) was used to summarize the differences among species and trait values of the most abundant and other co‐occurring subdominant species. Species from the Polytrichaceae were separated out on both PCA axes, indicating their high chlorophyll concentrations and photosynthetic capacities (axis 1) and relatively high‐light requirements (axis 2). Mniaceae species also had relatively high photosynthetic capacities, but their light saturation points were low. In contrast, *Racomitrium joseph‐hookeri* and *Lepidozia reptans*, two species with a high shoot mass per area, had high‐light requirements and low nutrient and chlorophyll concentrations and photosynthetic capacities. The nutrient concentrations, photosynthetic capacities, and photosynthetic nutrient use efficiencies of the most abundant bryophyte species did not differ from co‐occurring subdominant species. Our research confirms the links between the photosynthesis‐related traits and adaptation strategies of bryophytes. However, species relative abundance was not related to these traits.

## 1 INTRODUCTION

Although there are more than 25,000 bryophyte species in the world (Crum, 2001), their importance and functions in ecosystems are often underestimated or neglected because of their small size. However, their relatively simple structures and poikilohydric characteristics permit them to inhabit or even dominate in some environments where vascular plants are disadvantaged, such as peatlands, subalpine forest floors, and arctic regions (Glime, 2007; Vanderpoorten & Goffinet, 2009). In moist subalpine forests, bryopytes are abundant forest‐floor components and play important roles in energy flows, nutrient cycling, and water and soil conservation, as well as contributing considerably to biological diversity (Chapin, Oechel, Vancleve, & Lawrence, 1987; Lindo & Gonzalez, 2010; Liu & Bao, 2006). Although often regarded as a homogeneous “bryophyte mat,” bryophtye communities can be quite diverse both taxonomically and physiologically. However, in contrast to the taxonomic diversity, the physiological diversity of bryophyte communities has hardly been studied to date. Comparisons of physiological traits (such as the nutrient concentrations, chlorophyll concentrations, and photosynthetic capacities) of different bryophyte species living in the the same community can help to elucidate their different ecological functions, understand the resource use of the whole community, and contributes to a better prediction of community structure dynamics (Bona, Fyles, Shaw, & Kurz, 2013; Gunnarsson, Malmer, & Rydin, 2002; Modrzyński, Chmura, & Tjoelker, 2015).

Physiological traits have been found to relate to bryophyte architectural structures as well as to habitat conditions. For example, Marschall and Proctor (2004) found that bryophyte species from shady habitats and those in the Polytrichaceae family had relatively high mass‐based chlorophyll concentrations (Chl_mass_) compared to typical “sun” species. Waite and Sack (2010) reported that epiphytic mosses (receiving higher irradiance) possessed higher area‐based light‐saturated assimilation rates (A_area_), light compensation and saturation points (LCP and LSP), and mass‐based dark respiration rates (Rd_mass_) than terricolous species. And Wang, Liu, and Bao (2016) showed that the mean values of Chl_mass_, mass‐based light‐saturated assimilation rate (A_mass_), and photosynthetic nitrogen/phosphorus use efficiencies (PNUE and PPUE) of erect bryophyte species were significantly higher than those of prostrate species. However, the variation between species is large and the general patterns of trait‐value differences between functional groups does not apply to all species.

Subalpine understory bryophyte species include large pleurocarpous species (“feathermosses” such as *Hylocomium splendens* and *Pleurozium schreberi*), some of which are very abundant and widespread on the forest floor. Other large pleurocarps, however, only occupy discreet patches, as do the other subdominant species (Fenton & Bergeron, 2013; Vanderpoorten & Goffinet, 2009). Due to large biomass differences, the very abundant and the co‐occurring subdominant species will obviously differ in their contribution to ecosystem processes. What factors control species abundances and distributions within the forest is not clear, however. Several studies have investigated the effects of both biotic (e.g., vascular plant productivity) and abiotic (envrionmental conditions) factors on species compositions and abundance patterns in boreal‐forest understory bryophyte communities. Evans, Halpern, and McKenzie (2012) found that bryophyte cover and richness were only weakly related to overstorey structure or vascular plant abundance, and Fenton and Bergeron (2013) suggested that stochastic processes dominate the assembly of the bryophyte community. On the other hand, Jonsson et al. (2015) reported that moss communities were primarily influenced by vascular plant community composition and productivity, while abiotic factors played indirect roles.

Besides possibly being influenced by the vascular plant community and environmental conditions, bryophyte communities will be shaped by species interactions which, in turn, may be controlled by species characteristics such as the carbon and nutrient acquisition rates, nutrient‐use efficiencies, size, and architecture of the composing species (Freschet, Kichenin, & Wardle, 2015; Gong et al., 2011; Grime, 2002). Thereby, growth form appears to be an important factor, determining the growth potential through controlling hydration dynamics and determining competitive strengths through the potential to overgrow other species (Bates, 1998; Belote & Weltzin, 2006; Wang et al., 2016). The latter appears to be important in explaining the high abundance of some larger pleurocarpous forest‐floor species. However, not all large pleurocaps are equally abundant and the additional importance of physiological traits affecting observed abundance patterns is not clear to date. Previous studies have suggested that species abundance in a particular habitat is likely coupled with physiological advantages given local conditions, for example, high Chl_mass_ and photosynthetic capacities in light‐limited environments (Glime, 2007; Hájek, Tuittila, Ilomets, & Laiho, 2009). However, whether such advantages can help explain species relative abundances within communities is unknown. Comparisons of functional traits between the most abundant with all other co‐occurring subdominant understory bryophyte species will help us to answer this question and to better understand bryophyte community assembly.

We re‐analyzed the dataset from Wang, Liu, Bader, Feng, and Bao (2017) on functional traits of 27 bryophyte species in a temperate‐zone subalpine forest on the eastern Tibetan Plateau, studying the grouping of bryophyte species in the functional‐trait space using principal component analysis (PCA). We explored differences between taxa and tried to explain the most prominent differences based on the species' ecology and morphology. Moreover, the positions of the three most abundant species, each with >20% cover, and other co‐occurring subdominant species (<5% cover) were analyzed specifically. We hypothesized that the most abundant species should have higher nutrient concentrations, photosynthetic capacities, and/or photosynthetic nutrient use efficiencies compared to other bryophytes.

## 2 MATERIALS AND METHODS

### 2.1 Study site, focal species, sampling, and pre‐treatment

The study was conducted in an old‐growth fir forest in Dagu Glacier Park of Heishui County in Sichuan Province, China (102°46′E, 32°14′N, elevation between 3,640 and 3,655 m). The climate is characterized by dry, cold winters and short, wet summers. The mean annual temperature is 4.4°C and the mean annual precipitation is 620 mm. The forest has a canopy (42% coverage) dominated by *Abies fargesii* var. *faxoniana* and the common co‐occurring forest‐floor shrub and herbaceous species are *Rosa omeiensis*,* Ligularia sagittal*, and *Fragaria orientalis*. The understory is dominated by mosses (with a near‐80% ground cover), in particular *Actinothuidium hookeri*,* H. splendens*, and *Ptilium crista‐castrensis* (covering approximately 25%, 25%, and 20% of the ground area, respectively). Including these three most abundant species, 27 terricolous or saxicolous and saprolignicolous bryophyte species were sampled (two liverworts and 25 moss species; the vegetative shoot and reproductive shoot of *Mnium spinosum* were considered as two different morphotypes and sampled individually because of their distinct appearances, so we had 28 bryophyte types) (Table 1, Appendix S1). The nomenclature followed *Flora Bryophytorum Sinicorum* and the specimens were deposited in the herbarium at the Chengdu Institute of Biology, Chinese Academy of Sciences (collection number: Hei‐Bryo01 to Hei‐Bryo28).

**Table 1:** Twenty‐seven bryophyte species collected from the old‐growth fir forest of Dagu Glacier Park, China, and used for determining photosynthesis‐related functional traits. The category of life forms followed Mägdefrau (1982)

Scientific name	Code	Family	Lifeform	Habitat
Liverwort				
*Lepidozia reptans*	Lr	Lepidoziaceae	Mat	Rotten wood
*Scapania rotundifolia*	Sr	Scapaniaceae	Mat	Rock
Moss				
*Sphagnum junghuhnianum*	Sj	Sphagnaceae	Turf	Soil
*Campylopus schwarzii*	Cs	Dicranaceae	Turf	Rock
*Paraleucobryum enerve*	Pe	Dicranaceae	Turf	Soil
*Oncophorus wahlenbergii*	Ow	Dicranaceae	Cushion	Rock
*Racomitrium joseph‐hookeri*	Rj	Grimmiaceae	Cushion	Rock
*Rhizomnium nudum*	Rn	Mniaceae	Turf	Soil
*Mnium spinosum* (reproductive shoot)	Ms‐R	Mniaceae	Turf	Soil
*M. spinosum* (vegetative shoot)	Ms‐V	Mniaceae	Turf	Soil
*Plagiomnium japonicum*	Pj	Mniaceae	Weft	Soil
*Bartramia halleriana*	Bh	Bartramiaceae	Turf	Rock
*Leucodon morrisonensis*	Lm	Leucodontaceae	Tail	Trunk
*Thuidium kanedae*	Tk	Thuidiaceae	Weft	Soil
***Actinothuidium hookeri***	**Ah**	**Thuidiaceae**	**Weft**	**Soil**
*Climacium dendroides*	Cd	Climaciaceae	Dendroid	Soil
*Sanionia uncinata*	Su	Amblystegiaceae	Weft	Soil
*Entodon concinnus*	Ec	Entodontaceae	Weft	Rotten wood
*Plagiothecium handelii*	Ph	Plagiotheciaceae	Mat	Soil
*Heterophyllium affine*	Ha	Sematophyllaceae	Weft	Rotten wood
*Hypnum callichroum*	Hc	Hypnaceae	Weft	Soil
***Ptilium crista‐castrensis***	**Pc**	**Hypnaceae**	**Weft**	**Soil**
*Rhytidium rugosum*	Rr	Hylocomiaceae	Weft	Rock
*Rhytidiadelphus triquetrus*	Rt	Hylocomiaceae	Weft	Soil
*Pleurozium schreberi*	Ps	Hylocomiaceae	Weft	Soil
***Hylocomium splendens***	**Hs**	**Hylocomiaceae**	**Weft**	**Soil**
*Pogonatum microstomum*	Pm	Polytrichaceae	Turf	Soil
*Polytrichastrum alpinum*	Pa	Polytrichaceae	Turf	Soil

The bold text indicates the three most abundant species. The vegetative and reproductive shoots of *M. spinosum* were sampled separately because of their different appearances, see Appendix S1.

Detailed investigation methods and the mean trait values of each species have been published in Wang et al. (2016). Samples were collected between 10 a.m. and 5 p.m. in August 2012, which had a mean monthly temperature of 14°C (recorded by the temperature data loggers [DS1923 iButton, Maxim Integrated Products] on the top of the bryophyte canopies, with an interval of 30 min, *n *= 5 loggers). Four samples of each species were obtained from separated patches (at least 10 m apart). The bryophytes were collected with the underlying substrate, sealed in plastic bags and brought to the laboratory within 1 hr. We carefully removed the litter, bark, and other mixed mosses, eliminated dead tissues and only kept green sections as the final sample. After that, all of the samples were washed with distilled water to clean the dust and mud. From each sample, 25 g were oven‐dried at 70°C for 48 hr, ground to fine powder (60 mesh screen, 0.250 mm) and stored at −4°C for chemical analysis.

### 2.2 Measurements of gas exchange and mass per unit area

CO_2_‐exchange was measured using a Li‐Cor 6400‐22 L with a Lighted Conifer Chamber (Li‐Cor, Inc., Lincoln, NE, USA) in the laboratory. Detailed experimental methods for the photosynthetic light‐response curves measurement of bryophytes are also described in Wang et al. (2016). The samples were submerged in distilled water for 1 min, and the residual water on the surface of the tissue was carefully removed with a paper towel. They were then arranged into a Petri dish, mimicking their natural positions (i.e., weft‐forming mosses lying down, turf‐forming mosses standing up) but avoiding overlap between shoots. To prevent desiccation during the gas exchange measurement, moist water‐absorbing cotton was placed on the bottom of each dish (Romero, Putz, & Kitajima, 2006). Following 30 min of light induction under 150 μmol photons m^−2^ s^−1^ photosynthetically active radiation (PAR), the bryophyte tissues were transferred to the cuvette‐chamber with 15°C block temperature, 60%–80% relative humidity, a 400 ppm CO_2_ and a relatively low flow rate of 300 μmol/s to decrease the water loss and increase the CO_2_ signal. According to trial experiments, 12 steps of light intensity were set: 800, 600, 400, 300, 200, 150, 100, 80, 60, 40, 20, and 0 μmol m^−2^ s^−1^ PAR. Each light level lasted for about 3 min for the assimilation rate to reach a relatively steady state.

The experimental conditions of the photosynthetic CO_2_‐response curves were similar to those of the light‐response curves, except that the light intensities were fixed to be slightly higher than their light saturation points (LSP). According to a trial experiment, 11 steps of CO_2_ concentrations were set: 400, 300, 200, 100, 50, 200, 400, 600, 800, 1,000, and 1,200 ppm. Each CO_2_ level lasted for about 3 min for the assimilation rate to reach a relatively steady state.

A photograph of each sample was taken from directly above the bryophytes in their arrangement for the gas exchange measurements in the Petri dishes. ImageJ software (National Institutes of Health, USA) was used to calculate the projected shoot or leaf area (Waite & Sack, 2010) from these photographs. Light compensation point (LCP), LSP, area‐based photosynthetic and dark respirations rates (A_area_ and Rd_area_), and CO_2_ compensation point (CO_2_CP) were estimated by fitting a non‐rectangular hyperbola photosynthetic model (Ye, 2007) to the photosynthetic light‐/CO_2_‐response data, the fitting coefficients being all above 0.99. After the gas exchange measurement, the samples were oven‐dried at 70°C for 48 hr to determine the dry mass. Shoot mass per area (SMA) was derived as the dry mass divided by the projected area. Mass‐based photosynthetic and dark respiration rates (A_mass_ and Rd_mass_) were determined by dividing A_area_ and Rd_area_ by SMA.

### 2.3 Chemical analysis

Mass‐based carbon and nitrogen concentrations (C_mass_ and N_mass_) were measured using high temperature combustion by Vario Macro Cube Elemental Analyser (Elementar Analysensysteme GmbH, Germany) and mass‐based phosphorus concentration (P_mass_) was analyzed using the Mo‐Sb Antispectrophotography Method (Liu, 1996). Area‐based concentrations (C_area_, N_area_, and P_area_) were derived by multiplying the mass‐based concentrations and SMA. The stoichiometric ratios of C:N, C:P, and N:P were also calculated from the mass‐based concentrations. Photosynthetic nitrogen and phosphorus use efficiencies (PNUE and PPUE) were calculated by dividing A_mass_ by N_mass_ and P_mass_, respectively.

Chloroplast pigments were extracted from the liquid‐nitrogen‐preserved samples in the dark with 95% alcohol overnight (Shu, Zhang, Chen, Chen, & Xu, 2010). Concentrations of chlorophyll a and chlorophyll b were determined following the method of Bao and Leng (2005). Mass‐based chlorophyll concentration (Chl_mass_) was calculated as the sum of chlorophyll a and chlorophyll b. Area‐based concentrations (Chl_area_) were derived by multiplying Chl_mass_ by SMA. Light‐saturated assimilation rate per chlorophyll (A_chl_) was calculated by dividing A_mass_ by Chl_mass_.

### 2.4 Data analysis

Principal component analysis was used to explore associations between the 10 measured traits (including SMA, A_mass_, Rd_mass_, LSP, LCP, CO_2_CP, C_mass_, N_mass_, P_mass_, and Chl_mass_) and their distribution among species. Pearson and Spearman (when normality assumption not satisfied) correlations were determined for all pairwise combinations of traits among species. The independent‐samples *t*‐test or Mann–Whitney *U*‐test (normality assumption not satisfied) was used to test for differences in functional trait values between the three most abundant and the other, subordinate species. All of the statistical analyses were performed in PASW Statistics 19.0 (IBM, NY, USA) and Microcal Origin 9.0 (Northampton, MA, USA). Statistical results were considered significant when *p *≤ .05.

## 3 RESULTS

The first three PCA components accounted for 71% of the total variance (component 1 explained 35% and component 2 18%) (Table 2). Axis 1 corresponded to a combined gradient of general activity (nutrient and chlorophyll concentrations and CO_2_‐exchange potential), and an inverse gradient of SMA. Axis 2 corresponded mainly to CO_2_‐exchange parameters, with more light‐adapted characteristics (high A_mass_, high LCP, and LSP) to the top and more shade‐adapted characteristics at the bottom. As is clear from the PCA and from the correlation analysis (Appendix S2), many of the parameters are strongly correlated.

**Table 2:** Results from a principal component analysis of functional traits of 27 bryophyte species (28 types) from the old‐growth fir forest of Dagu Glacier Park, China. Shown are values of component loadings and final communality extractions and the percent of variance explained by each component

Trait	Component 1	Component 2	Component 3	Communality extraction
SMA	−**0.753**	0.142	−0.188	0.642
C_mass_	−0.158	0.359	**0.705**	0.724
N_mass_	**0.816**	−0.234	−0.204	0.775
P_mass_	**0.732**	−0.432	−0.162	0.791
Chl_mass_	**0.722**	0.444	−0.093	0.758
A_mass_	**0.538**	**0.622**	−0.302	0.854
Rd_mass_	**0.651**	**0.585**	0.070	0.794
LCP	−0.043	**0.515**	0.324	0.891
LSP	−0.402	0.312	−**0.534**	0.732
CO_2_CP	**0.577**	−0.376	0.414	0.784
% variance explained	35.245	18.194	12.672	

LSP, light saturation points; SMA, shoot mass per area.

Bold values indicate loadings which were considered valid for the component.

**Figure 1:**
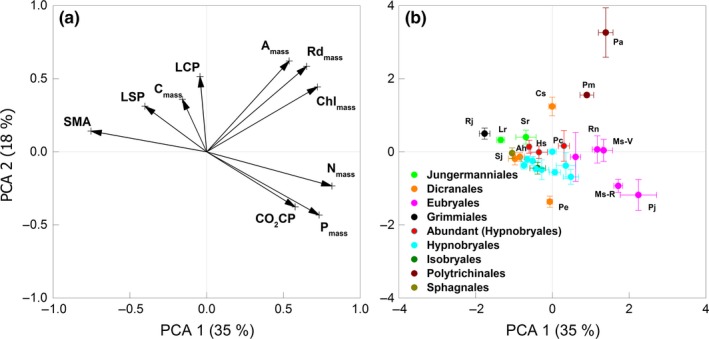
Principal component analysis of bryophyte functional traits of 27 species (28 types) collected from the subalpine old‐growth fir forest of Dagu Glacier Park, China. (a) Loading plots of the studied functional traits. The traits include shoot mass per area (SMA), mass‐based light‐saturated assimilation and dark respiration rates (A_mass_ and Rd_mass_), concentrations of carbon, nitrogen, phosphorus, and chlorophyll (C_mass_, N_mass_, P_mass_, and Chl_mass_), light saturation and compensation points (LSP and LCP), and CO_2_ compensation point (CO_2_ CP). (b) Loading plots of the 28 bryophyte species. The codes of species names are defined in Table 1. Different colors indicate the taxonomic groups and the three most abundant species (in red)

The farthest species in the PCA‐reduced trait space, in the high‐activity and light‐adapted corner were the two Polytrichaceae, *Polytrichastrum alpinum* and, to a lesser degree, *Pogonatum microstomum* (Figure 1b), which had the highest mean values of A_mass_, Rd_mass_, and Chl_mass_ (Appendix S3–S5). The Eubryales, especially the Mniaceae species also had high “activity” scores (axis 1), corresponding to relatively high A_mass_, Rd_mass_, CO_2_CP, N_mass_, P_mass_, and Chl_mass_, while their SMA and LSP were low. On the low‐activity, high‐density end of the first PCA axis were *Racomitrium joseph‐hookeri* and *Lepidozia reptans*, with their relatively high SMA, LSP, and LCP. The Hypnobryales (including the three most abundant species) were central on both axes, whereas Dicranales were quite central on the first axis but spread widely on the second axis. The three most abundant species were not separated from those co‐occurring in the trait space (Figure 1b). The values of A_chl_ of the most abundant species were higher than those of the subordinate species (Table 3), whereas other parameters (N_mass_, P_mass_, Chl_mass_, A_mass_, Rd_mass_, PNUE, and PPUE) did not differ.

**Table 3:** Comparisons of functional trait values (mean ± *SE*, range of species means and *p* values for comparisons) between the three most abundant (A) and 24 co‐occurring (C) subdominant bryophyte species collected from the subalpine old‐growth fir forest of Dagu Glacier Park, China. One species (*Mnium spinosum*) was treated as two species, resulting in 25 datasets for the co‐occurring species (see Appendix S1)

Traits	Symbol	Units	Group	Mean ± *SE*	Range	*p*
Nitrogen per mass	N_mass_	%	A	0.92 ± 0.03	0.87–0.96	.198^T^
			C	1.17 ± 0.06	0.74–1.98	
Phosphorus per area	P_mass_	%	A	0.12 ± 0.00	0.12–0.13	.794^U^
			C	0.13 ± 0.01	0.07–0.31	
Chlorophyll per mass	Chl_mass_	mg/g	A	0.73 ± 0.26	0.29–1.20	.270^U^
			C	1.78 ± 0.31	0.15–5.51	
Light‐saturated assimilation rate per mass	A_mass_	nmol CO_2_ g^−1^ s^−1^	A	14.43 ± 5.16	6.58–24.16	.869^U^
			C	16.20 ± 3.58	3.27–71.96	
Dark respiration per mass	Rd_mass_	nmol CO_2_ g^−1^ s^−1^	A	15.68 ± 2.97	11.88–21.53	.758^U^
			C	13.70 ± 2.15	4.40–52.59	
Photosynthetic nitrogen use efficiency	PNUE	nmol CO_2_ (g N)^−1^ s^−1^	A	1.73 ± 0.65	0.68–2.92	.647^U^
			C	1.38 ± 0.28	0.36–6.17	
Photosynthetic phosphorus use efficiency	PPUE	nmol CO_2_ (g P)^−1^ s^−1^	A	11.76 ± 3.48	5.46–17.46	.831^U^
			C	13.83 ± 3.27	3.60–67.81	
Light‐saturated assimilation rate per chlorophyll	A_chl_	nmol CO_2_ (g Chl)^−1^ s^−1^	A	20.27 ± 1.54	18.04–23.58	**.025** ^U^
			C	11.79 ± 2.04	2.08–52.38	

Mean values and standard error are based on the mean of bryophyte type.

Independent samples *t*‐test (^T^) or Mann–Whitney *U*‐test (^U^, normal assumption not satisfied) was used to test differences in the mean values for the abundant and co‐occurring subdominant bryophyte species. Bold value indicates the significant difference between the two groups ( *p* ≤ .05).

## 4 DISCUSSION

### 4.1 Explaining species traits

The PCA axis 1 and the correlation results confirmed the trait trade‐offs between nutrient concentrations, photosynthetic rates, and construction cost in bryophytes (Shipley, Lechowicz, Wright, & Reich, 2006; Wang, Bao, Feng, & Lin, 2014; Wright et al., 2004). The high Chl_mass_, A_mass_, and nutrient photosynthetic use efficiencies of the Polytrichaceae in comparison with other species agree with previous research (Marschall & Proctor, 2004). This high photosynthetic potential can be attributed to their structural characteristics. First, the highly specialized hydrome and leptome of Polytrichaceae allows a relatively high hydraulic conductivity and efficient resource redistribution within the plants (Atala & Alfaro, 2012). Moreover, in most species, lamellae on the upper leaf surface increase the surface area for CO_2_ uptake and prevent water loss (Proctor, 2005). These evolved characteristics contribute to the high photosynthetic capacities and efficiencies of Polytrichaceae bryophytes (Figure 1b), making them a versatile bryophyte group that can be shade‐tolerant, but also well adapted to high‐light environments and can even become the pioneer species in clear‐cut habitats (Bao, 2005). However, the studied Polytrichaceae species (unlike some other species in this family) can grow to a height of only about 10 cm and can grow laterally only by sending up new shoots from below, not by overgrowing from the top, as the larger pleurocarps can (Appendix S6). This gives them a competitive disadvantage that, together with their relatively high‐light requirements, may explain why these species, in spite of their physiological potential, are not especially abundant in our forest although in disturbed forests and plantations they can become quite abundant (Leuschner & Ellenberg, 2017; Wang, Bao, Yan, & Lin, 2014).

The high Chl_mass_ (the highest in this study) in the four Mniaceae species (Appendix S5) probably represents their adaptation to shade, permitting them to absorb light efficiently and resulting in a relatively high A_mass_ (Appendix S3). These species also showed particularly low values of SMA and LSP, reflecting the adaptive value in shady environments of investing in a large light‐capturing area and a saving in the capacity of photosynthetic dark reactions (Klinka, Krajina, Ceska, & Scagel, 1989; Marschall & Proctor, 2004). In contrast, *R. joseph‐hookeri* and *L. reptans* had the lowest scores on the first PCA axis, reflecting their denser morphology and resource‐conservative ecological strategy (Figure 1b) (Wright et al., 2004). *Racomitrium joseph‐hookeri* forms cushions made up of small, erect individuals, and *L. reptans* a forms richly branched and intertwining mats (Appendix S6). Both species have high tissue thickness and densitiy per unit area, and possessed the highest SMA among the studied species. As a result, their N_mass_, P_mass_, and Chl_mass_ were relatively low while the area‐based values were relatively high. Dense packing of shoots or thalli plays a crucial role in controlling water loss, but compromises tissue light interception (Bates, 1998). Because the upper layer of the densely packed photosynthetic tissues may block the light from the deeper layers, fewer nutrients will be invested in photosynthetic capacities in these layers. This thus limits the photosynthetic rates of the whole moss shoot, even if arranged without self‐shading, as in our experiment (Rice, Aclander, & Hanson, 2008; Rice, Neal, Mango, & Black, 2011; Zotz, Schweikert, Jetz, & Westerman, 2000). As a result, A_mass_ of these compact species was very low and the LCP and LSP were relatively high.

The coexistence of bryophytes with different adaptive strategies on the studied forest floor may indicate environmental heterogeneity, but it does not have to. The big question on the mechanisms of species coexistence (Hubbell, 2001; Schimper, 1902), mostly tested for vascular plants, to our knowledge remains unexplored for bryophyte communities: to what extent do the functional differences indicate the need for habitat heterogeneity and/or niche partitioning, and to what extent are species ecologically equivalent and can coexistence be explained by “neutral” processes like chance establishment?

### 4.2 The most abundant species do not have physiological advantageous compared to subordinate species

There were no significant differences in photosynthesis‐related functional traits between the mean values of the most abundant species and other bryophytes in the studied subalpine forest (Figure 1b, Table 3, Appendix S3–S5). The first explanation of the apparent unimportance of photosynthetic traits may be that long‐term carbon gain is not regulated primarily via the potential rates. Our gas exchange measurements were conducted under optimal environmental conditions for the studied species and describe the potential photosynthetic capacities. In the actual growing sites, the ambient environment is continually changing and usually not optimal for photosynthesis. Thus, the actual carbon gain and growth potential may depend more on the amount of time the bryophytes can maintain a positive net photosynthesis than merely on a high photosynthetic capacity. This activity time depends crucially on the bryophyte's water uptake, storage, and retention capacities (Wagner, Bader, & Zotz, 2014; Zotz et al., 2000).

Second, rather than growing fast, other intrinsic factors may be more important for the current abundant species to adapt and utilize the limited resource of the subalpine forest understory. For example, the growth form determines whether a bryophyte can overgrow competitors (vascular and non‐vascular) or is easily overgrown. The most abundant bryophytes in the studied forest have their current year branches grow on last year branches. They thus form new levels and “climb” over other bryophytes, small vascular plants, and fresh litter (Liu, Wang, Bao, & Li, 2015). This growth form might be the reason that these large pleurocarpous mosses can dominate the floors of boreal and subalpine forests because the resulting dense and continuous carpet can restrict the growth of other bryophytes through changing soil temperatures, lowering the already‐low light levels, and by intercepting nutrient deposition (Cleavitt, 2004; Startsev, Lieffers, & McNabb, 2007). However, in spite of the intuitive importance of growth form, this alone cannot explain the dominance in the current study site because, for example, *P. schreberi* and *Rhytidium rugosum* have a similar growth form and size as the most abundant species, but are far less abundant in the same community.

## 5 CONCLUSION

In summary, we found that some taxonomic groups, especially the Polytrichaceae and Mniaceae, stood out among the other bryophytes with their photosynthesis‐related traits, apparently reflecting different morphologies and adaptation strategies. The three most abundant bryophyte species did not differ in their physiological traits from other co‐occurring subdominant bryophytes. Understanding the role of photosynthesis and other processes for bryophyte growth and community composition still requires a lot of further research. By describing the distribution of traits within a bryophyte community, we hope to allow future comparisons as such descriptions from other habitats become available.

## CONFLICT OF INTEREST

None declared.

## AUTHOR CONTRIBUTIONS

ZW and WB designed the research; ZW and XL collected the data; ZW and ZZ analyzed the data, and ZW, MB, and XL wrote the manuscript.
