# Supplementary material for: Comparisons of photosynthesis‐related traits of 27 abundant or subordinate bryophyte species in a subalpine old‐growth fir forest
Source: Ecol Evol. 2017 Aug 11;7(18):7454–61. doi: 10.1002/ece3.3277 (PMC5606851; doi:10.1002/ece3.3277)
Supplement: Supplementary file 1 [file ECE3-7-7454-s001.docx]

# Appendix

Wang et al. Comparisons of photosynthesis-related traits of 27 abundant or subordinate bryophyte species in a subalpine old-growth fir forest

**Appendix S1.** Comparisons of trait values between reproductive and vegetative shoots of *Mnium spinosum.*

The morphologies of reproductive shoot and vegetative shoot of *Mnium spinosum* are quite different: the reproductive shoots are erect, leaves spirally inserted, forming rosettes; and the vegetative shoots are arcuate, leaves spirally-arranged. The trait values of the current study were similar for both bryophyte shoots, except that the vegetative shoots possessed higher Rd_mass_ and Rd_area_ than those of the reproductive shoots, and the latter had higher CO_2_CP (Appendix S3). Which indicate that the vegetative shoots are more metabolic active and more efficient in CO_2_ diffusion, probably due to their different leaf arrangements.

**Appendix S2** Bivariate correlations among mass-based saturated assimilation rates (A_mass_), dark respiration rates (Rd_mass_), concentrations of chlorophyll (Chl_mass_), carbon (C_mass_), nitrogen (N_mass_) and phosphorus (P_mass_), shoot mass per area (SMA), photosynthetic light compensation points (LCP), photosynthetic light saturation points (LSP) and photosynthetic CO_2_ compensation points (CO_2_CP) of 27 bryophyte species (28 bryophyte types) collected from the old growth fir forest of Dagu Glacier, China (n=4 for each bryophyte type).

| Trait | A_mass_ | Rd_mass_ | Chl_mass_ | N_mass_ | P_mass_ | SMA | LCP | LSP | CO_2_CP | C_mass_ |
| --- | --- | --- | --- | --- | --- | --- | --- | --- | --- | --- |
| A_mass_ | — |  |  |  |  |  |  |  |  |  |
| Rd_mass_ | 0.582^***^ | — |  |  |  |  |  |  |  |  |
| Chl_mass_ | 0.608^***^ | 0.614^***^ | — |  |  |  |  |  |  |  |
| N_mass_ | 0.274^**^ | 0.312^**^ | 0.549^***^ | — |  |  |  |  |  |  |
| P_mass_ | 0.280^**^ | 0.385^***^ | 0.372^***^ | 0.699^***^ | — |  |  |  |  |  |
| SMA | -0.489^***^ | -0.655^***^ | -0.591^***^ | -0.523^***^ | -0.601^***^ | — |  |  |  |  |
| LCP | -0.013 | 0.346^***^ | 0.063 | -0.132 | -0.167 | 0.114 | — |  |  |  |
| LSP | -0.029 | -0.100 | -0.159 | -0.196^*^ | -0.236^*^ | 0.400^***^ | 0.202^*^ | — |  |  |
| CO_2_CP | -0.065 | 0.223^*^ | 0.158 | 0.472^***^ | 0.504^***^ | -0.466^***^ | 0.098 | -0.328^***^ | — |  |
| C_mass_ | -0.035 | 0.054 | 0.067 | -0.242^*^ | -0.313^**^ | 0.073 | 0.145 | -0.118 | -0.040 | — |

Methods of Pearson correlation and Spearman correlation (when normal distribution was not satisfied) were used, p ≤ 0.05.**Appendix S3** The mean values (Mean ± SE ) of shoot/leaf mass per area (SMA/LMA), mass- and area-based light-saturated assimilation rates and dark respiration rates (A_mass_, A_area_, Rd_mass_ and Rd_area_), Light saturation point (LSP), compensation point (LCP) and CO_2_ compensation point (CO_2_CP) of 27 bryophyte species (28 bryophyte types) collected from the old growth fir forest of Dagu Glacier, China (n=4 for each bryophyte type).

| Species | SMA  (g m^-2^)^+^ | A_mass_  (nmol CO_2_ g^-1^ s^-1^) | A_area_  (μmol CO_2_ m^-2^ s^-1^) | Rd_mass_  (nmol CO_2_ g^-1^ s^-1^) | Rd_area_  (μmol CO_2_ m^-2^ s^-1^) | LSP  (μmol photons m^-2^ s^-1^) | LCP  (μmol photons m^-2^ s^-1^) | CO_2_CP  (ppm CO_2_) |
| --- | --- | --- | --- | --- | --- | --- | --- | --- |
| *Lepidozia reptans* | 244.41 ± 14.47 | 3.27 ± 0.69 | 0.80 ± 0.17 | 4.40 ± 0.49 | 1.06 ± 0.07 | 534.87 ± 27.08 | 104.59 ± 14.53 | 196.51 ± 9.50 |
| *Scapania rotundifolia* | 113.26 ± 9.03 | 6.73 ± 0.77 | 0.78 ± 0.14 | 8.09 ± 0.30 | 0.92 ± 0.10 | 462.00 ± 50.83 | 51.97 ± 4.74 | 210.68 ± 37.32 |
| *Sphagnum junghuhnianum* | 175.77 ± 7.61 | 9.50 ± 1.29 | 1.69 ± 0.29 | 6.82 ± 1.12 | 1.21 ± 0.22 | 278.12 ± 58.25 | 32.99 ± 4.82 | 127.20 ± 11.19 |
| *Campylopus schwarzii* | 76.47 ± 4.93 | 25.69 ± 4.63 | 1.96 ± 0.35 | 24.74 ± 4.09 | 1.88 ± 0.29 | 348.89 ± 76.67 | 93.21 ± 9.30 | 200.96 ± 10.49 |
| *Paraleucobryum enerve* | 88.01 ± 7.03 | 7.99 ± 1.36 | 0.69 ± 0.11 | 7.53 ± 1.71 | 0.66 ± 0.14 | 361.59 ± 74.19 | 26.15 ± 6.22 | 257.40 ± 13.75 |
| *Oncophorus wahlenbergii* | 194.77 ± 10.40 | 11.29 ± 1.62 | 2.22 ± 0.40 | 5.97 ± 0.78 | 1.14 ± 0.09 | 437.28 ± 129.49 | 42.86 ± 7.24 | 163.72 ± 9.25 |
| *Racomitrium joseph-hookeri* | 330.34 ± 24.85 | 5.88 ± 0.37 | 1.95 ± 0.22 | 5.21 ± 0.52 | 1.69 ± 0.11 | 734.08 ± 155.35 | 74.92 ± 9.17 | 185.41 ± 10.28 |
| *Rhizomnium nudum* | 56.09 ± 8.59 | 10.60 ± 2.71 | 0.62 ± 0.23 | 15.30 ± 1.86 | 0.85 ± 0.16 | 266.96 ± 51.92 | 62.80 ± 19.53 | 230.10 ± 23.83 |
| *Mnium spinosum* (vegetative shoot) | 48.81 ± 4.95 | 20.50 ± 2.91 | 1.01 ± 0.21 | 26.32 ± 2.23 | 1.29 ± 0.19 | 225.86 ± 13.35 | 43.45 ± 13.48 | 300.12 ± 19.28 |
| *Mnium spinosum* (reproductive shoot) | 46.96 ± 3.28 | 14.80 ± 2.50 | 0.69 ± 0.11 | 16.53 ± 2.03 | 0.76 ± 0.07 | 199.83 ± 10.94 | 62.99 ± 6.65 | 360.88 ± 6.42 |
| *Plagiomnium japonicum* | 36.53 ± 4.55 | 28.42 ± 3.43 | 1.06 ± 0.21 | 31.73 ± 5.09 | 1.10 ± 0.10 | 342.93 ± 70.27 | 38.66 ± 14.52 | 308.84 ± 43.03 |
| *Bartramia halleriana* | 50.76 ± 4.38 | 29.79 ± 2.19 | 1.52 ± 0.19 | 15.18 ± 5.65 | 0.76 ± 0.31 | 298.35 ± 89.71 | 54.42 ± 45.91 | 297.62 ± 22.49 |
| *Leucodon morrisonensis* | 129.21 ± 9.18 | 7.19 ± 0.92 | 0.91 ± 0.10 | 5.60 ± 0.59 | 0.72 ± 0.10 | 415.84 ± 92.30 | 45.62 ± 7.68 | 174.87 ± 15.18 |
| *Thuidium kanedae* | 54.44 ± 4.57 | 13.32 ± 1.68 | 0.70 ± 0.04 | 15.46 ± 4.41 | 0.89 ± 0.33 | 327.84 ± 80.27 | 44.28 ± 12.32 | 242.49 ± 42.15 |
| ***Actinothuidium hookeri*** | **123.14 ± 5.07** | **6.58 ± 0.64** | **0.81 ± 0.08** | **13.61 ± 1.30** | **1.68 ± 0.18** | **498.79 ± 26.27** | **72.29 ± 12.73** | **198.43 ± 11.30** |
| *Climacium dendroides* | 123.85 ± 13.87 | 10.05 ± 2.25 | 1.18 ± 0.22 | 6.47 ± 1.32 | 0.75 ± 0.08 | 543.06 ± 49.74 | 31.51 ± 1.59 | 167.31 ± 19.48 |
| *Sanionia uncinata* | 101.65 ± 5.36 | 4.70 ± 1.12 | 0.48 ± 0.12 | 9.37 ± 0.95 | 0.96 ± 0.13 | 407.39 ± 56.99 | 66.68 ± 4.55 | 211.18 ± 13.42 |
| *Entodon concinnus* | 80.58 ± 5.00 | 17.43 ± 4.85 | 1.33 ± 0.28 | 14.36 ± 1.05 | 1.16 ± 0.11 | 590.52 ± 61.92 | 38.73 ± 3.06 | 148.46 ± 7.57 |
| *Plagiothecium handelii* | 83.87 ± 6.80 | 5.97 ± 0.71 | 0.49 ± 0.05 | 11.71 ± 1.20 | 0.96 ± 0.02 | 452.44 ± 105.07 | 84.95 ± 10.42 | 226.32 ± 7.80 |
| *Heterophyllium affine* | 117.13 ± 5.53 | 5.30 ± 1.18 | 0.63 ± 0.15 | 8.57 ± 1.02 | 0.99 ± 0.09 | 460.79 ± 98.96 | 72.09 ± 13.50 | 238.74 ± 6.95 |
| *Hypnum callichroum* | 71.99 ± 8.65 | 6.66 ± 1.61 | 0.46 ± 0.11 | 10.39 ± 1.44 | 0.74 ± 0.12 | 503.67 ± 59.88 | 57.79 ± 12.58 | 222.60 ± 3.73 |
| ***Ptilium crista-castrensis*** | **46.49 ± 7.19** | **24.16 ± 10.34** | **1.04 ± 0.36** | **21.53 ± 8.91** | **0.94 ± 0.36** | **305.09 ± 38.88** | **68.46 ± 18.78** | **240.53 ± 12.56** |
| *Rhytidium rugosum* | 140.06 ± 4.38 | 9.86 ± 1.09 | 1.37 ± 0.11 | 8.52 ± 0.99 | 1.19 ± 0.15 | 499.31 ± 109.97 | 51.21 ± 5.49 | 141.59 ± 8.15 |
| *Rhytidiadelphus triquetrus* | 110.79 ± 8.99 | 3.79 ± 0.83 | 0.41 ± 0.08 | 8.85 ± 2.12 | 0.94 ± 0.18 | 422.44 ± 77.77 | 67.52 ± 9.76 | 201.41 ± 14.55 |
| *Pleurozium schreberi* | 130.29 ± 11.78 | 5.78 ± 1.01 | 0.79 ± 0.18 | 5.66 ± 0.49 | 0.73 ± 0.09 | 296.79 ± 55.69 | 46.51 ± 10.21 | 189.52 ± 4.37 |
| ***Hylocomium splendens*** | **104.03 ± 10.22** | **12.54 ± 2.01** | **1.32 ± 0.25** | **11.88 ± 3.45** | **1.33 ± 0.50** | **341.98 ± 63.14** | **73.07 ± 14.23** | **205.74 ± 12.54** |
| *Pogonatum microstomum* | 93.68 ± 8.08 | 68.41 ± 5.73 | 6.54 ± 1.09 | 17.06 ± 1.73 | 1.56 ± 0.06 | 525.53 ± 88.09 | 30.85 ± 3.36 | 127.69 ± 10.09 |
| *Polytrichastrum alpinum* | 98.11 ± 14.55 | 71.96 ± 1.87 | 7.01 ± 0.97 | 52.59 ± 8.45 | 5.24 ± 1.38 | 422.03 ± 138.26 | 99.04 ± 33.82 | 184.38 ± 5.19 |

A similar table has also been published in Wang *et al.* (2016). The **bold** text indicates the three most abundant species.**Appendix S4** The mean values (Mean ± SE ) of mass- and area-based carbon, nitrogen and phosphorus concentrations (C_mass_, C_area_, N_mass_, N_area_, P_mass_ and P_area_) and the stoichiometric ratios (C:N, C:P and N:P) of 27 bryophyte species (28 bryophyte types) collected from the old growth fir forest of Dagu Glacier, China (n=4 for each bryophyte type).

|  | C_mass_  (%) | C_area_  (g m^-2^) | N_mass_  (%) | N_area_  (g m^-2^) | P_mass_  (%) | P_area_  (g m^-2^) | C:N | C:P | N:P |
| --- | --- | --- | --- | --- | --- | --- | --- | --- | --- |
| *Lepidozia reptans* | 36.55 ± 0.30 | 89.23 ± 4.75 | 0.87 ± 0.07 | 2.12 ± 0.22 | 0.08 ± 0.01 | 0.19 ± 0.02 | 42.95 ± 3.36 | 480.56 ± 37.84 | 11.20 ± 0.30 |
| *Scapania rotundifolia* | 40.99 ± 0.30 | 46.42 ± 3.67 | 1.00 ± 0.13 | 1.10 ± 0.06 | 0.07 ± 0.01 | 0.08 ± 0.00 | 42.74 ± 4.54 | 618.19 ± 72.14 | 14.46 ± 0.62 |
| *Sphagnum junghuhnianum* | 38.07 ± 0.28 | 66.96 ± 3.26 | 0.88 ± 0.05 | 1.54 ± 0.07 | 0.08 ± 0.00 | 0.14 ± 0.01 | 43.84 ± 2.66 | 470.67 ± 10.52 | 10.84 ± 0.61 |
| *Campylopus schwarzii* | 36.95 ± 0.35 | 28.26 ± 1.89 | 0.79 ± 0.05 | 0.61 ± 0.08 | 0.07 ± 0.00 | 0.05 ± 0.00 | 47.67 ± 3.51 | 547.89 ± 23.34 | 11.67 ± 0.97 |
| *Paraleucobryum enerve* | 34.42 ± 0.30 | 30.24 ± 2.21 | 1.11 ± 0.06 | 0.99 ± 0.12 | 0.14 ± 0.01 | 0.12 ± 0.02 | 31.21 ± 1.99 | 256.71 ± 28.55 | 8.16 ± 0.41 |
| *Oncophorus wahlenbergii* | 36.27 ± 0.12 | 72.37 ± 4.65 | 0.98 ± 0.17 | 1.93 ± 0.35 | 0.08 ± 0.00 | 0.16 ± 0.00 | 40.24 ± 8.72 | 443.80 ± 18.07 | 11.86 ± 2.00 |
| *Racomitrium joseph-hookeri* | 36.94 ± 0.20 | 122.06 ± 9.40 | 0.74 ± 0.05 | 2.42 ± 0.11 | 0.09 ± 0.01 | 0.31 ± 0.04 | 50.66 ± 3.99 | 410.44 ± 44.41 | 8.31 ± 1.15 |
| *Rhizomnium nudum* | 37.60 ± 0.18 | 21.06 ± 3.17 | 1.50 ± 0.07 | 0.99 ± 0.05 | 0.18 ± 0.03 | 0.10 ± 0.02 | 25.19 ± 1.07 | 224.45 ± 31.67 | 8.80 ± 0.94 |
| *Mnium spinosum* (vegetative shoot) | 38.15 ± 0.42 | 18.58 ± 1.79 | 1.41 ± 0.16 | 0.71 ± 0.13 | 0.16 ± 0.02 | 0.08 ± 0.01 | 28.59 ± 4.35 | 242.48 ± 30.29 | 8.63 ± 0.76 |
| *Mnium spinosum* (reproductive shoot) | 37.67 ± 0.09 | 17.70 ± 1.26 | 1.86 ± 0.12 | 0.87 ± 0.07 | 0.22 ± 0.03 | 0.10 ± 0.01 | 20.56 ± 1.41 | 185.20 ± 29.73 | 9.38 ± 2.17 |
| *Plagiomnium japonicum* | 34.09 ± 0.99 | 12.46 ± 1.61 | 1.98 ± 0.22 | 0.71 ± 0.10 | 0.31 ± 0.05 | 0.11 ± 0.02 | 18.11 ± 2.75 | 123.59 ± 26.87 | 6.66 ± 0.45 |
| *Bartramia halleriana* | 37.42 ± 0.48 | 19.02 ± 1.76 | 1.24 ± 0.05 | 0.62 ± 0.04 | 0.12 ± 0.01 | 0.06 ± 0.00 | 30.42 ± 1.42 | 314.04 ± 22.11 | 10.31 ± 0.41 |
| *Leucodon morrisonensis* | 38.94 ± 0.30 | 50.28 ± 3.42 | 0.87 ± 0.02 | 1.12 ± 0.07 | 0.11 ± 0.03 | 0.14 ± 0.03 | 45.03 ± 0.87 | 403.76 ± 76.50 | 8.91 ± 1.58 |
| *Thuidium kanedae* | 36.81 ± 0.88 | 20.05 ± 1.84 | 1.24 ± 0.15 | 0.66 ± 0.05 | 0.18 ± 0.01 | 0.10 ± 0.01 | 31.34 ± 4.36 | 208.78 ± 10.07 | 7.15 ± 1.26 |
| ***Actinothuidium hookeri*** | **38.31 ± 0.06** | **47.18 ± 1.96** | **0.96 ± 0.04** | **1.18 ± 0.06** | **0.12 ± 0.01** | **0.15 ± 0.01** | **40.24 ± 1.50** | **322.30 ± 17.51** | **8.02 ± 0.44** |
| *Climacium dendroides* | 35.13 ± 1.29 | 43.07 ± 3.47 | 1.15 ± 0.16 | 1.39 ± 0.18 | 0.13 ± 0.01 | 0.15 ± 0.01 | 32.05 ± 3.60 | 283.73 ± 15.57 | 9.03 ± 0.54 |
| *Sanionia uncinata* | 35.37 ± 0.37 | 35.94 ± 1.87 | 1.05 ± 0.08 | 1.08 ± 0.11 | 0.10 ± 0.01 | 0.10 ± 0.01 | 34.04 ± 2.28 | 359.27 ± 20.93 | 10.69 ± 0.87 |
| *Entodon concinnus* | 35.68 ± 0.29 | 28.79 ± 1.95 | 1.20 ± 0.06 | 0.96 ± 0.04 | 0.15 ± 0.01 | 0.12 ± 0.01 | 29.88 ± 1.58 | 241.35 ± 15.38 | 8.20 ± 0.82 |
| *Plagiothecium handelii* | 35.88 ± 0.92 | 31.81 ± 2.09 | 1.52 ± 0.17 | 1.33 ± 0.06 | 0.15 ± 0.02 | 0.13 ± 0.01 | 24.13 ± 2.62 | 245.33 ± 35.37 | 10.15 ± 0.81 |
| *Heterophyllium affine* | 35.68 ± 0.30 | 41.76 ± 1.74 | 1.11 ± 0.16 | 1.32 ± 0.24 | 0.13 ± 0.01 | 0.15 ± 0.02 | 34.16 ± 4.72 | 290.10 ± 27.24 | 8.86 ± 1.10 |
| *Hypnum callichroum* | 34.80 ± 0.32 | 25.10 ± 3.13 | 1.25 ± 0.07 | 0.91 ± 0.14 | 0.13 ± 0.01 | 0.09 ± 0.01 | 27.99 ± 1.23 | 269.00 ± 13.00 | 9.71 ± 0.81 |
| ***Ptilium crista-castrensis*** | **36.42 ± 0.30** | **17.00 ± 2.78** | **0.87 ± 0.06** | **0.39 ± 0.04** | **0.13 ± 0.01** | **0.06 ± 0.01** | **42.71 ± 3.39** | **289.27 ± 30.75** | **6.89 ± 0.93** |
| *Rhytidium rugosum* | 36.68 ± 0.17 | 51.39 ± 1.80 | 1.22 ± 0.06 | 1.70 ± 0.07 | 0.14 ± 0.02 | 0.20 ± 0.02 | 30.27 ± 1.46 | 264.58 ± 29.33 | 8.70 ± 0.75 |
| *Rhytidiadelphus triquetrus* | 36.91 ± 0.35 | 40.95 ± 3.58 | 0.85 ± 0.05 | 0.93 ± 0.06 | 0.11 ± 0.01 | 0.12 ± 0.01 | 44.07 ± 2.71 | 369.44 ± 54.26 | 8.27 ± 0.80 |
| *Pleurozium schreberi* | 37.24 ± 0.35 | 48.59 ± 4.64 | 0.77 ± 0.05 | 1.00 ± 0.13 | 0.10 ± 0.01 | 0.13 ± 0.00 | 49.13 ± 2.97 | 370.26 ± 29.58 | 7.63 ± 0.82 |
| ***Hylocomium splendens*** | **37.28 ± 0.40** | **38.76 ± 3.72** | **0.94 ± 0.17** | **0.98 ± 0.20** | **0.12 ± 0.02** | **0.12 ± 0.02** | **44.46 ± 8.67** | **345.89 ± 57.66** | **7.98 ± 0.52** |
| *Pogonatum microstomum* | 35.86 ± 0.86 | 33.46 ± 2.42 | 1.45 ± 0.11 | 1.36 ± 0.13 | 0.12 ± 0.00 | 0.11 ± 0.01 | 24.97 ± 1.37 | 293.01 ± 5.44 | 11.82 ± 0.58 |
| *Polytrichastrum alpinum* | 37.87 ± 0.51 | 37.08 ± 5.40 | 1.21 ± 0.14 | 1.19 ± 0.24 | 0.11 ± 0.01 | 0.11 ± 0.02 | 32.46 ± 3.77 | 355.44 ± 23.80 | 11.28 ± 1.15 |

A similar table has also been published in Wang *et al.* (2016). The **bold** text indicates the three most abundant species.**Appendix S5** The mean values (Mean ± SE ) of mass- and area-based chlorophyll pigments (Chl_mass_ and Chl_area_), ratio of chlorophyll a : chlorophyll b (Chl a:b), photosynthetic nitrogen, phosphorus and chlorophyll use efficiencies (PNUE, PPUE and A_Chl_) of 27 bryophyte species (28 bryophyte types) collected from the old growth fir forest of Dagu Glacier, China (n=4 for each bryophyte type).

|  | Chl_mass_  (mg g^-1^) | Chl_area_  (g m^-2^) | Chl a:b | PNUE  [nmol CO_2_ (g N)^-1^ s^-1^] | PPUE  [nmol CO_2_ (g P)^-1^ s^-1^] | A_Chl_  [nmol CO_2_ (g Chl)^-1^ s^-1^] |
| --- | --- | --- | --- | --- | --- | --- |
| *Lepidozia reptans* | 0.56 ± 0.18 | 0.14 ± 0.06 | 1.90 ± 0.19 | 4.00 ± 1.05 | 45.08 ± 12.26 | 8.37 ± 3.17 |
| *Scapania rotundifolia* | 1.34 ± 0.25 | 0.14 ± 0.01 | 2.06 ± 0.23 | 7.22 ± 1.31 | 104.93 ± 20.71 | 5.75 ± 1.30 |
| *Sphagnum junghuhnianum* | 0.53 ± 0.09 | 0.09 ± 0.02 | 1.27 ± 0.13 | 11.04 ± 1.70 | 118.47 ± 18.64 | 18.70 ± 2.21 |
| *Campylopus schwarzii* | 2.45 ± 0.15 | 0.19 ± 0.01 | 1.51 ± 0.10 | 33.60 ± 7.36 | 377.57 ± 59.81 | 10.44 ± 1.66 |
| *Paraleucobryum enerve* | 0.15 ± 0.01 | 0.01 ± 0.00 | 1.48 ± 0.30 | 7.22 ± 1.17 | 59.94 ± 11.38 | 52.38 ± 6.60 |
| *Oncophorus wahlenbergii* | 0.63 ± 0.13 | 0.12 ± 0.03 | 1.72 ± 0.24 | 11.69 ± 2.25 | 135.23 ± 30.85 | 19.72 ± 3.52 |
| *Racomitrium joseph-hookeri* | 0.56 ± 0.17 | 0.18 ± 0.05 | 1.27 ± 0.10 | 8.04 ± 0.71 | 66.61 ± 11.88 | 13.97 ± 3.80 |
| *Rhizomnium nudum* | 5.51 ± 0.50 | 0.30 ± 0.04 | 1.60 ± 0.06 | 7.16 ± 1.99 | 62.48 ± 18.46 | 2.08 ± 0.67 |
| *Mnium spinosum* (vegetative shoot) | 3.97 ± 0.39 | 0.20 ± 0.03 | 1.38 ± 0.08 | 15.47 ± 3.14 | 134.86 ± 31.23 | 5.35 ± 0.89 |
| *Mnium spinosum* (reproductive shoot) | 3.35 ± 0.44 | 0.16 ± 0.02 | 1.51 ± 0.03 | 7.87 ± 1.00 | 77.18 ± 24.03 | 4.46 ± 0.54 |
| *Plagiomnium japonicum* | 2.18 ± 0.29 | 0.08 ± 0.02 | 1.10 ± 0.11 | 14.60 ± 1.65 | 98.98 ± 17.16 | 13.21 ± 1.12 |
| *Bartramia halleriana* | 1.50 ± 0.12 | 0.08 ± 0.01 | 1.63 ± 0.06 | 24.42 ± 2.76 | 251.43 ± 29.43 | 20.14 ± 1.72 |
| *Leucodon morrisonensis* | 0.34 ± 0.07 | 0.04 ± 0.01 | 1.42 ± 0.08 | 8.33 ± 1.12 | 74.23 ± 16.84 | 22.57 ± 3.90 |
| *Thuidium kanedae* | 1.17 ± 0.08 | 0.06 ± 0.01 | 1.28 ± 0.10 | 10.79 ± 0.52 | 76.31 ± 11.64 | 11.55 ± 1.62 |
| ***Actinothuidium hookeri*** | **0.29 ± 0.04** | **0.03 ± 0.00** | **1.52 ± 0.12** | **6.84 ± 0.44** | **54.61 ± 3.16** | **23.58 ± 2.10** |
| *Climacium dendroides* | 1.62 ± 0.35 | 0.20 ± 0.04 | 1.29 ± 0.05 | 8.49 ± 1.03 | 77.50 ± 12.14 | 6.42 ± 1.08 |
| *Sanionia uncinata* | 0.54 ± 0.07 | 0.06 ± 0.01 | 1.13 ± 0.03 | 4.40 ± 0.94 | 47.90 ± 12.02 | 8.42 ± 1.41 |
| *Entodon concinnus* | 1.82 ± 0.20 | 0.15 ± 0.01 | 1.70 ± 0.03 | 14.18 ± 3.54 | 119.88 ± 34.30 | 9.42 ± 1.87 |
| *Plagiothecium handelii* | 1.82 ± 0.27 | 0.16 ± 0.03 | 1.23 ± 0.02 | 3.62 ± 0.54 | 36.14 ± 4.25 | 3.81 ± 1.22 |
| *Heterophyllium affine* | 0.91 ± 0.21 | 0.11 ± 0.03 | 1.74 ± 0.06 | 4.89 ± 1.21 | 40.38 ± 5.59 | 6.10 ± 1.00 |
| *Hypnum callichroum* | 1.59 ± 0.47 | 0.11 ± 0.02 | 1.33 ± 0.08 | 5.29 ± 1.26 | 50.34 ± 11.17 | 4.43 ± 0.84 |
| ***Ptilium crista-castrensis*** | **1.20 ± 0.11** | **0.06 ± 0.01** | **1.51 ± 0.07** | **29.21 ± 12.63** | **174.59 ± 57.97** | **21.48 ± 10.05** |
| *Rhytidium rugosum* | 0.58 ± 0.06 | 0.08 ± 0.01 | 2.13 ± 0.12 | 8.05 ± 0.70 | 70.48 ± 10.41 | 17.11 ± 0.38 |
| *Rhytidiadelphus triquetrus* | 0.58 ± 0.21 | 0.06 ± 0.02 | 1.09 ± 0.02 | 4.39 ± 0.71 | 36.01 ± 5.71 | 9.29 ± 3.00 |
| *Pleurozium schreberi* | 0.80 ± 0.26 | 0.10 ± 0.04 | 1.07 ± 0.04 | 7.56 ± 1.26 | 59.55 ± 12.94 | 11.13 ± 5.54 |
| ***Hylocomium splendens*** | **0.71 ± 0.07** | **0.07 ± 0.01** | **1.48 ± 0.04** | **15.82 ± 5.43** | **123.63 ± 40.41** | **18.04± 2.94** |
| *Pogonatum microstomum* | 5.02 ± 0.19 | 0.47 ± 0.04 | 1.22 ± 0.07 | 47.73 ± 4.73 | 559.81 ± 49.74 | 13.66 ± 1.11 |
| *Polytrichastrum alpinum* | 4.91 ± 0.38 | 0.48 ± 0.08 | 1.37 ± 0.08 | 61.66 ± 7.12 | 678.08 ± 59.74 | 14.86 ± 0.88 |

A similar table has also been published in Wang *et al.* (2016). The **bold** text indicates the three most abundant species.

Appendix S6 Photos of 14 of the studied bryophyte species from the old growth fir forest of Dagu Glacier, China.

| *Lepidozia*  *reptans* | 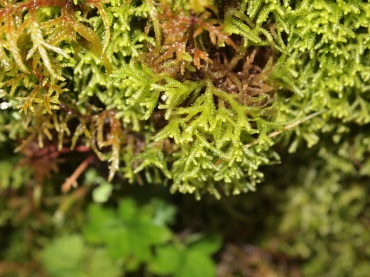 | ***Actinothuidium***  ***hookeri*** | 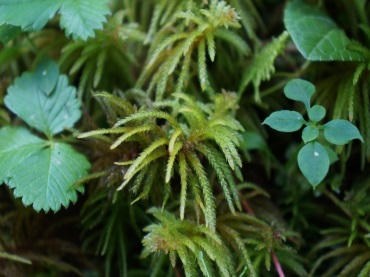 |
| --- | --- | --- | --- |
| *Sphagnum*  *junghuhnianum* | 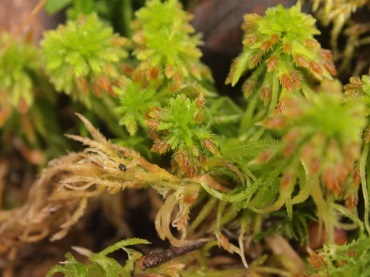 | ***Ptilium***  ***crista-castrensis*** | 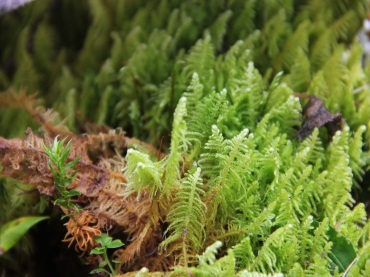 |
| *Campylopus*  *schwarzii* | 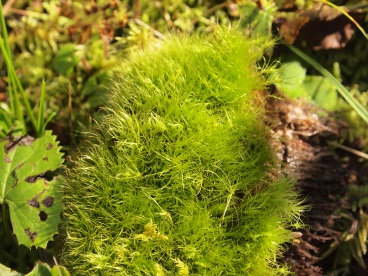 | *Rhytidiadelphus*  *triquetrus* | 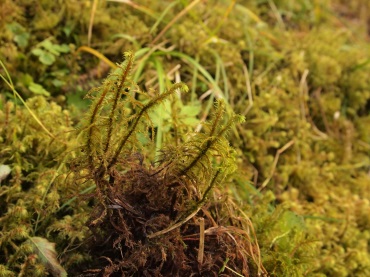 |
| *Racomitrium*  *joseph-hookeri* | 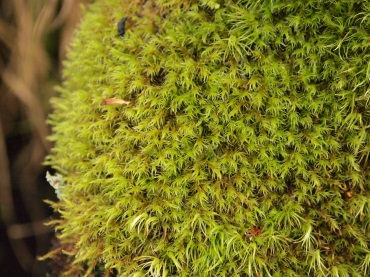 | *Pleurozium*  *schreberi* | 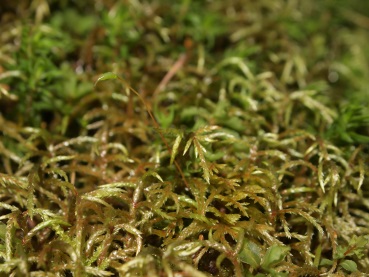 |
| *Rhizomnium*  *nudum* | 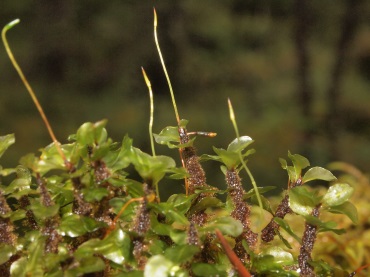 | ***Hylocomium***  ***splendens*** | 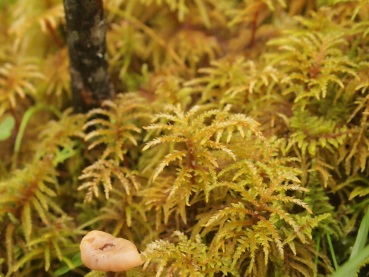 |
| *Mnium*  *spinosum* | 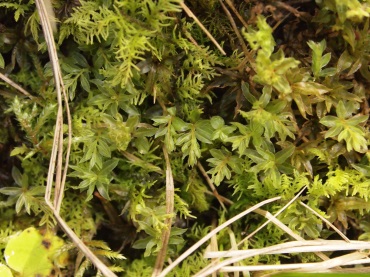 | *Pogonatum*  *microstomum* | 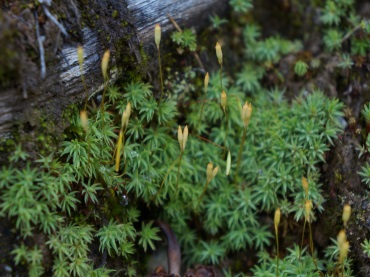 |
| *Bartramia*  *halleriana* | 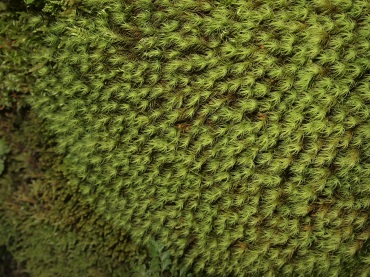 | *Polytrichastrum*  *alpinum* | 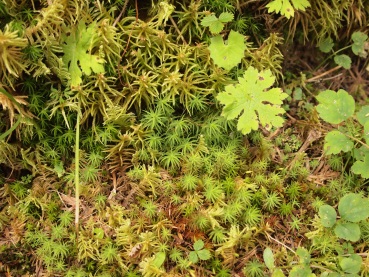 |

The **bold** text indicates the three most abundant species.
